# The Approach of Pregnant Women to Vaccination Based on a COVID-19 Systematic Review

**DOI:** 10.3390/medicina57090977

**Published:** 2021-09-17

**Authors:** Sławomir M. Januszek, Anna Faryniak-Zuzak, Edyta Barnaś, Tomasz Łoziński, Tomasz Góra, Natalia Siwiec, Paweł Szczerba, Rafał Januszek, Tomasz Kluz

**Affiliations:** 1 Department of Gynecology and Obstetrics, Fryderyk Chopin University Hospital No. 1, 35-055 Rzeszów, Poland; natalia_s@opoczta.pl (N.S.); jtkluz@interia.pl (T.K.); 2 Department of Gynecology and Obstetrics, University Hospital No. 2, 35-029 Rzeszów, Poland; ania.ef2@gmail.com; 3 Department of Gynecology and Obstetrics, Institute of Medical Sciences, Medical College of Rzeszow University, 35-310 Rzeszow, Poland; ebarnas@interia.eu; 4 Department of Gynecology and Obstetrics, Clinical Specialist Hospital Profamilia, 35-302 Rzeszów, Poland; tomasz.lozinski@profamilia.pl (T.Ł.); minddin@gmail.com (T.G.); 5 Department of Gynecology and Obstetrics, Jan Paweł II Hospital, 35-241 Reszów, Poland; 6 Department of Gynecology and Obstetrics, Jagiellonian University Medical College, 31-501 Kraków, Poland; pawelszczerba@poczta.fm; 7 Department of Clinical Rehabilitation, University of Physical Education, 31-571 Kraków, Poland; jaanraf@interia.pl

**Keywords:** COVID-19 vaccination, pregnancy, acceptance, hesitancy, attitude, intention to undergo vaccination

## Abstract

*Background and Objectives*: Pregnant women are more likely to develop a more severe course of COVID-19 than their non-pregnant peers. There are many arguments for the safety and efficacy of COVID-19 vaccines in pregnant women. The aim of this study is to conduct a systematic review concerning the approach of pregnant women towards vaccination against COVID-19, with particular regard to determinants of vaccination acceptance. *Materials and Methods*: Articles were reviewed in which the aim was to evaluate—via a survey or questionnaire—the acceptance and decision to undergo vaccination against COVID-19. The articles were subjected to review according to recommendations of Preferred Reporting Items for Systematic Reviews and Meta-Analyses Statement (PRISMA). *Results:* In various studies, the percentage of pregnant women accepting the COVID-19 vaccine was between 29.7% and 77.4%. The strongest factors co-existing with the acceptance of the COVID-19 vaccination in pregnancy were trust in the importance and effectiveness of the vaccine, explicit communication about the safety of COVID-19 vaccines for pregnant women, acceptance of other vaccinations such as those for influenza, belief in the importance of vaccines/mass vaccination in one’s own country, anxiety about COVID-19, trust in public health agencies/health science, as well as compliance to mask guidelines. The remaining factors were older age, higher education, and socioeconomic status. *Conclusions:* This review allowed us to show that geographic factors (Asian, South American countries) and pandemic factors (different threats and risks from infection) significantly influence the acceptance of vaccines. The most significant factors affecting acceptance are those related to public awareness of the risk of infection, vaccine safety, and the way in which reliable information about the need and safety of vaccines is provided. Professional and reliable patient information by obstetricians and qualified medical personnel would significantly increase the level of confidence in vaccination against COVID-19.

## 1. Introduction

The coronavirus disease 2019 (COVID-19) pandemic has led to many deaths worldwide [1]. Massive loss of human life has created public health challenges, overburdened health systems, disrupted supply chains and the economy, while triggering a mental health crisis [2,3]. Pregnant and postpartum women may be more likely to develop a more severe course of COVID-19 than their non-pregnant peers [4,5,6,7,8]. The concept of severe disease includes a course requiring hospitalization, intensive care, ventilator usage, special respiratory equipment, and/or death. In some reports, it has been suggested that severe acute respiratory syndrome coronavirus 2 (SARS-CoV-2) infection in pregnant women is associated with an increased risk of severe disease course, with the need for invasive ventilation or extracorporeal membrane oxygenation, and/or even death [5,6,7,8,9]. In another study, a link has been found between COVID-19 and the risk of pre-term and cesarean deliveries [10]. The cases of vertical transmission of the virus [11], which could lead to hydrops fetalis and death of the fetus have also been reported [12]. Children are more susceptible to asymptomatic infections; however, they are carriers of SARS-COV-2, transmitting the virus to others, including pregnant women [13,14]. Pregnant women affected by COVID-19 are at risk of premature birth and show an increased risk of other adverse pregnancy outcomes, compared to pregnant women without COVID-19 [1]. It is also worth emphasizing that the COVID-19 pandemic causes fear for the health of the fetus and one’s own health among pregnant women, which significantly affects their well-being [15].

### 1.1. COVID-19 Vaccination during Pregnancy

Several vaccines have been developed and approved for use while maintaining the appropriate regulations [16,17]. In a review on the safety of vaccinations during pregnancy, Pratama et al. noted Pfizer-BioNTech and Moderna vaccines to be effective in preventing infection while being considered safe for pregnancy and the fetus [18]. Vaccines can slow down the epidemic if vaccination is widely accepted. There are many arguments in favor of COVID-19 vaccine safety. These vaccines contain mRNA encapsulated in a lipid nanoparticle that is transferred into cells. The host cells produce coronavirus spike proteins that stimulate the formation of antibodies. This process occurs robustly in regional lymph nodes [19]. Noteworthy is the fact that none of the COVID-19 vaccines contain live viruses or adjuvants that may affect a developing fetus. A reproductive and developmental toxicity study in rats administered the Moderna mRNA vaccine did not show any alarming signals regarding the safety of female reproduction, intrauterine, or postnatal development [20]. In another study, evidence supporting the safety and efficacy of Pfizer BNT162b2 in pregnancy was provided, showing health benefits for both the mother and newborn [21]. COVID-19 vaccination may be associated with a fever typically lasting up to 2 days. Increased temperature is not unusual in pregnancy, and it can be successfully lowered with acetaminophen. Further studies on vaccination during pregnancy will provide data on both vaccine safety concerns and vaccine efficacy for mothers and newborns. Information for healthcare providers on its use in counseling and for patients has been developed [22,23].

### 1.2. Acceptance of COVID-19 Vaccination during Pregnancy

It is necessary to understand factors influencing the acceptance of vaccinations among various social groups, including pregnant women, which will significantly contribute to the return of society to a pre-pandemic state [24]. Pregnant women often play a key role in whether their children will receive vaccinations; however, the results obtained by Skjefte et al. showed the differential COVID-19 vaccine acceptance level among pregnant women and its similarity with previous reports from the general population [1]. The World Health Organization (WHO) posted reluctance to vaccinate as one of the greatest health threats in the world even before the COVID-19 pandemic [25]. In preliminary research on the acceptance of COVID-19 vaccines, unprecedented challenges were predicted for vaccination on a global scale [26,27,28]. The Advisory Committee on Immunization Practices and the American College of Obstetricians and Gynecologists (ACOG) recommends that pregnant women be vaccinated. The role of obstetricians in encouraging pregnant women to vaccinate is also emphasized. The Polish Society of Gynecologists and Obstetricians based on the analysis of data published around the world and the authors’ own research and observations according to the position of ACOG, College of Obstetricians and Gynecologists (RCOG), Centers for Disease Control and Prevention (CDC), and Society for Maternal–Fetal Medicine (SMFM), recommends COVID-19 vaccines for pregnant and lactating women [29]. 

### 1.3. Objective

The aim of this study was to conduct a systematic review regarding the approach of pregnant women toward vaccination against COVID-19, with particular regard to determinants of COVID-19 vaccination acceptance.

## 2. Methodology

### 2.1. Search Strategy and Study Selection

The recommendations of Preferred Reporting Items for Systematic Reviews and Meta-Analyses Statement (PRISMA) were used in this review. Articles published in PubMed in which the aim was to evaluate acceptance, decision to vaccinate against COVID-19–carried out via a survey or questionnaire—were eligible for inclusion in this review. The search was performed for studies published until 10 July 2021, using the following strategy: (COVID * pregnancy * vaccine * hesitancy [Title/Abstract]), OR (COVID * pregnancy * vaccine * acceptance [Title/Abstract]), OR (COVID * pregnancy * vaccine * hesitancy [Title/Abstract])), OR (COVID * pregnancy * intention to vaccinate * [Title/Abstract]), OR (COVID * pregnancy * vaccine * acceptance [Title/Abstract]), OR (COVID * pregnancy * vaccine * attitude [Title/Abstract] AND (2020:2021 [pdat]).

### 2.2. Eligibility and Inclusion Criteria

The inclusion criteria were as follows: peer-reviewed published articles indexed in PubMed; survey studies among the population of pregnant women; the major aim of the study was to evaluate COVID-19 vaccine acceptance or hesitancy among the population of pregnant women; publication language was English.

### 2.3. Exclusion Criteria

The exclusion criteria were as follows: unpublished manuscripts; publication language other than English; in the article, the aim was not an evaluation of COVID-19 vaccine acceptance or hesitancy among the population of pregnant women.

### 2.4. Data Extraction

Two researchers independently identified articles from PubMed as recommended by PRISMA. The third researcher verified and compared the extracted data. Titles, abstracts, and full articles were reviewed, and data were collected for the following items: date of the study, the country where the study was conducted, the target population of the study, i.e., pregnant women, the total number of respondents, and acceptance of the COVID-19 vaccine.

## 3. Results

**Figure 1:**
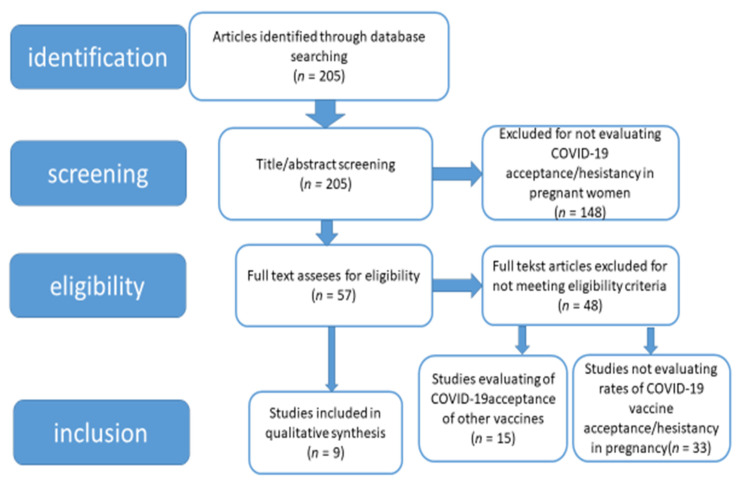
PRISMA flow chart of studies screened and included.

The articles selected for revision were aimed at evaluating acceptance and the decision to vaccinate against COVID-19-via a survey or questionnaire (Figure 1).

### 3.1. Characteristics of Articles in Presented Review

A total of nine published articles were analyzed in this review. These studies included pregnancy acceptance surveys of the COVID-19 vaccine from a total of 24 different countries. The studies were most often conducted in the United Kingdom (*n* = 3), followed by France, the United States, Switzerland, and Ireland (*n* = 2 for each country). The studies were carried out on dates ranging from June 2020 to February 2021. Some of them were performed in several or a dozen countries, including the study by Skjefte et al. [1] covering 16 countries and the study by Ceulemans et al. [30] covering 6 European countries. The largest sample size (17,871) for the international study with an online survey was conducted by Skjefte et al. [1], and the smallest (152) was found in the case of the Turkish study by Gencer et al. [31] among pregnant women who had at least primary school education, were above the age of 18, and voluntarily participated in the trial. In total, 6 of the analyzed studies used 20 questionnaires addressed to pregnant women, while the remaining 3 studies used 8 questionnaires addressed to pregnant and breastfeeding mothers. The majority of the studies were conducted between October 2020 and February 2021 (Table 1).

### 3.2. COVID-19 Vaccine Acceptance among Pregnant Women

The COVID-19 vaccine acceptance in various studies, presented as a percentage of pregnant and breastfeeding mothers was between 29.7% and 77.4%. Classified by study and country, the highest vaccine acceptance rate among pregnant women was observed in the research conducted by Tao et al. [33] in China (77.4%), Mohan et al. [37] in Qatar (75%), and in Italy (74.5%) by Mappa et al. [34]. In an online study by Skjefte et al. [1] carried out among pregnant women, 52.0% (*n* = 2747) intended to receive the COVID-19 vaccination during pregnancy. Responses of pregnant women varied significantly from country to country (range: 28.8–84.4%). In the current study, the COVID-19 vaccine acceptance rate was over 80% for pregnant women in Mexico and India and less than 45% in the US, Australia, and Russia. In contrast, the lowest vaccine acceptance rates among pregnant women were found in the trial performed in Switzerland (29.7%) among pregnant and breastfeeding women by Suckelberger et al. [36], and in the study conducted by Geoghegan et al. [35] (38%) among pregnant women in Ireland. In seven out of nine studies, it was found that the vaccination acceptance rate among pregnant women exceeded 50%. In Table 2, the degree of vaccine acceptance is demonstrated for individual studies as well as the most common concerns related to vaccination and factors determining acceptance of vaccination in pregnancy.

The most common concerns among pregnant women were fear of harm to the fetus and the occurrence of side effects that could negatively affect the fetus. Noteworthy is the increased co-existence of lower socioeconomic status, younger age among pregnant women who refuse vaccination, as well as the suspicion that the introduction of vaccines and advertising campaigns are politically motivated. The strongest factors co-existing with the acceptance of the COVID-19 vaccination during pregnancy were trust in the importance and effectiveness of the vaccine, explicit communication about the safety of COVID-19 vaccines for pregnant women, acceptance of other vaccinations such as those for influenza, belief in the importance of vaccines/mass vaccination for one’s own country, anxiety about COVID-19, trust in public health agencies/health science, as well as compliance to mask guidelines. The remaining factors were older age, higher education, and higher socioeconomic status. 

### 3.3. Changes in Acceptance of COVID-19 Vaccine among Pregnant Women over Time

The level accepting vaccines against COVID-19 varied over time, from the level of approx. 29% for the study conducted in June and July in Switzerland [36] to the level of 77.4% for the study in China [33] or Qatar [37] totaling 75% in November or January, and in February, 74.5% for the Italian study [34]. 

## 4. Discussion

### 4.1. Pandemic Aspects

Reluctance to undergo vaccination is a well-known phenomenon that has become a serious threat, for example, due to the return of some infectious diseases in the form of measles or whooping cough epidemics [38,39,40,41]. The tremendous advances in the development of effective and safe vaccines against COVID-19 have been unprecedented in the short term [42,43]. Nevertheless, reluctance to vaccinate for COVID-19 may be a limiting step in global efforts to control the current pandemic, with its negative health and socio-economic impact [44]. Assessment of the level of population immunity necessary to limit the spread of the pathogen depends on the basal reproductive abundance of this infectious disease [45], while COVID-19 estimates allow the indication of a range between 60 and 75% of the immune population needed to contain virus transmission and spread in communities [46,47]. Efficacy and duration of protection are important factors in achieving population immunity [48]; however, rejection of vaccinations may be a critical factor in preventing control of the COVID-19 pandemic [49]. Vaccine acceptance rates can help plan actions and interventions needed to raise awareness and reassure people of the safety and benefits of vaccines, which, in turn, would help control the spread of the virus and mitigate the negative effects of this unprecedented pandemic [50,51]. Assessment of attitudes and acceptance rates toward COVID-19 vaccines can help in choosing the appropriate form of communication, necessary to strengthen confidence in vaccination [52]. Mothers often have the greatest influence on the decision to vaccinate children and other family members; thus, it is also important to measure trust and the most important predictors of vaccination acceptance among pregnant women, frequently, at the same time, mothers of young children [1]. Vaccine acceptance could be influenced by sociodemographic factors such as age, gender, demography, and income status, individual factors such as personal beliefs, political views, perception of risk, and social or organizational factors, such as social media or the role of authorities [53].

### 4.2. Sociodemographic, Geographic, and Pandemic Acceptance Factors for Vaccination against COVID-19

Although the review found large variability in vaccine acceptance rates against COVID-19, a pattern of acceptance among pregnant women can be noted. In East and Southeast Asia (India, China, Qatar) [1,33,37], and some South American countries (Brazil and Mexico) [1], as well as Italy [34], the acceptance rates of pregnant women relative to the general public were relatively high. In contrast, for countries in Europe [30], North America [1,32], Australia [1], and Russia [1], lower acceptance rates of around 50% were recorded. The percentage of vaccine acceptors was, therefore, geographically dependent, but also dependent on the timing of the pandemic development. In countries where the pandemic had significantly affected countries from the outset, such as China and Italy, a fairly high degree of acceptance among pregnant women was observed. Moreover, in the summer months, even before the introduction of the vaccine, a much lower degree of vaccine acceptance was noted than in the case of studies conducted during the second wave. This indirectly results from the fact that social assessment of the risk is of great importance in the acceptance of vaccinations among pregnant women. Changes in the level of acceptance among pregnant women during a pandemic may be related to both individual and social or organizational factors [53]. Increased perceived risk of infection, benefits of vaccines, government restrictions, penalties for not using masks, as well as intense communication of the threat from traditional and social media can have a significant impact on willingness to vaccinate [53]. In most of the analyzed studies, older age, higher education, and higher income were also associated with higher official acceptance of the vaccine [1,30,32,33,34,36]. Many factors affecting the acceptance of the COVID-19 vaccine, such as geographic or socioeconomic factors, are difficult to modify.

### 4.3. Modifiable Factors of Vaccination Acceptance among Pregnant Women

Some of the predictors of vaccine acceptance, such as level of confidence in health institutions promoting vaccines and level of awareness of COVID-19 among pregnant women, are largely correctable variables. Factors that recur in most of the studies included in the review are those related to the level of awareness of COVID-19 risks in pregnancy and the safety of vaccination during its duration. Factors such as trust in received information on vaccination [30], confidence in the safety and efficacy of the COVID-19 vaccine [1], belief in the importance of vaccines [1], trust in routine childhood vaccines [1], concern regarding the COVID-19 pandemic [1], trust in public health agencies, no fear of vaccine side effects [31], reliable information [35], explicit communication about the safety of COVID-19 vaccines for pregnant women [34], having an obstetrician supervising pregnancy [36], influenza vaccination within the previous year [35], as well as confidence in the safety of the COVID-19 vaccine [34], all seem to have a common denominator. These are factors that concern providing information and awareness to pregnant or breastfeeding women about the current state of knowledge on COVID-19, vaccination against COVID-19, or vaccination in general. Similar factors also have significant influences on the decision to undergo vaccination in other social groups and the general population [42]. Communication strategies can contain positive directions for action, including encouragement from close and trusted people such as doctors and religious leaders, sharing personal experiences, or peer pressure [51]. Taking the information gathered in the above review into account, it should also be emphasized that evidence-based professional ethics in obstetrics and gynecology provide clear guidance on vaccination [54,55].

### 4.4. Professional Counseling

There is evidence that a physician’s recommendation to vaccinate is the most important factor in maternal decision making, regardless of geographic or social context [56,57]. During the pandemic, the anxiety of pregnant women for the health of the fetus and their own health had a negative impact on their well-being [15]. Mortazavi et al. also suggest that perhaps by supporting pregnant women, health professionals–including midwives–could reduce the level of anxiety and thus improve well-being. Such support could also reduce the anxiety associated with vaccination against COVID-19. Reliable information from qualified medical personnel on the current state of knowledge regarding safety, effectiveness, and recommendations of scientific societies may contribute to wider acceptance of vaccination against COVID-19 among pregnant women. Rather than pointing to the risk of the disease itself when recommending a vaccine, public health campaigns focused on the protective role and safety of the vaccine among pregnant and breastfeeding women may prove beneficial [23].

### 4.5. Limitations

This review is subject to some limitations. Data taken from scientific databases may not provide the most up-to-date public opinion due to peer-review and publication processes. The publicly accessible PubMed database was used. The inclusion of the studies was not complete but contained a large number of major polls and important factors to provide a complete image of present trends.

Care should be taken when interpreting and using the results, as survey intentions or responses may not directly predict future behavior. Moreover, opinions may change, especially in a raging pandemic. Further tracking of vaccine susceptibility could reveal whether reported incidents or clinical trial results and, subsequently, the introduction of new vaccines or new methods of treatment will further change people’s minds about vaccines.

## 5. Conclusions

Reluctance toward vaccination is a direct threat in the fight against COVID-19, as achieving population immunity depends on the effectiveness of the vaccine itself and the readiness of the population to accept it. In this review, an analysis is offered regarding attitudes toward vaccination among pregnant and breastfeeding women. According to the literature, in these studies, there are demographic and socioeconomic divisions in absorptive capacity, and the bias of some indicators is unprecedented. Pregnant and breastfeeding women, as well as mothers of young children, often play key roles in the acceptance of vaccinations by their entire families. This review allows highlighting that geographic factors (Asian, South American countries) and pandemic factors (different threats and risks from infection) significantly influence the acceptance of vaccines. However, the most important factors affecting acceptance are those related to public awareness of the risk of infection, vaccine safety, and the way in which reliable information on the need for vaccination and safety is communicated. Professional and reliable information must be provided to patients by obstetricians and qualified medical personnel, which will further significantly increase the level of trust in anti-COVID-19 actions.

## Figures and Tables

**Table 1:** Characteristics of papers included in present review.

Study	Country	Date of Study	Type of Survey	*N*
Levy et al. [32]	United States	December 2020 to January 2021	During visit	662
Skjefte et al. [1]	United States, India, Brazil, Russia, Spain, Argentina, Colombia, UK, Mexico, Peru, South Africa, Italy, Chile, Philippines, Australia and New Zealand	October 2020 to November 2020	On-line survey	17,871
Ceulemans et al. [30]	Belgium, Norway, The Netherlands, Switzerland, Ireland and UK	June 2020 to July 2020	Online survey	16,063
Tao et al. [33]	China	November 2020	During visit	1392
Gencer et al. [31]	Turkey	July 2020 to October 2020	Online survey	152
Mappa et al. [34]	Italy	January 2021 to February 2021	During visit	161
Geoghegan et al. [35]	Ireland	January 2021	During visit	300
Stuckelberger et al. [36]	Switzerland	June 2020 to July 2020	Online survey	2064
Mohan et al. [37]	Qatar	October 2020 to November 2020	During visit	341

**Table 2:** Characteristics of vaccine acceptance among pregnant women.

Study, *N*	Target Population	Acceptance Rate (%)/Date of the Survey	Reasons for Vaccine Rejection	Factors Correlating with Greater Vaccine Acceptance
Levy et al., 662	pregnant women undergoing prenatal screening	58.3% 12.2020–01.2021	risk to the fetus or neonate (45.8%), vaccine side-effects (17.7%), not believing vaccines are safe (16.2%)	Strongest: Trust in information received about vaccinations. Others: Older age, higher education, acceptance of influenza vaccination.
Skjefte et al., 17,871	women aged 18 years or older, currently pregnant, or with at least 1 child below the age of 18	52.0% 10–11.2020	possible harmful side-effects for baby (65.9%), incomplete data on safety and efficacy in pregnant women (48.8%), approval of the vaccine would be rushed for political reasons (44.9%)	Strongest: Confidence in COVID-19 vaccine safety and efficacy, belief in the importance of vaccines, confidence in routine childhood vaccines, anxiety about COVID-19, trust in public health agencies, compliance to mask guidelines. Others: Older age, greater income, higher education level, marital status, and health insurance.
Ceulemans et al., 16,063	pregnant and breastfeeding women	52.0% 06–07.2020	concerns about the safety of vaccines, lower educational and employment level	Strongest: Higher educational and employment level.
Tao et al., 1392	pregnant women	77.4% 11.2020	concerns about fetus safety (45%) and side-effects (21%)	Strongest: High level of perceived cues for action, high level of perceived susceptibility, low level of perceived barriers, high level of perceived benefits. Others: Young age, western region, low level of education, late pregnancy, high knowledge score regarding COVID-19.
Gencer et al., 152	pregnant women who with at least primary school education, who were above the age of 18 and voluntarily participated in the study	52.6% 07–10.2020	negative news in the media (21.7%) stating that vaccinations are not safe, concern about the subject of side-effects (21.7%)	Strongest: Mid-level or high income, no concerns about the risk of vaccination side effects. Others: Thinking that vaccinations strengthen immunity, believing that the benefits of vaccination are greater than its risks.
Mappa et al., 161	pregnant women attending Ospedale Cristo Re Università Roma TorVergata	74.5% 01–02.2021	vaccine campaigns	Strongest: Reliable information. Others: Higher educational and employment levels.
Geoghegan et al., 300	women pregnant during the introduction of the PfizerBioNTech vaccine	38.0% 01.2021	concerns about fetus safety	Strongest: Communication about safety of COVID-19 vaccines for pregnant women explicitly.
Stuckelberger et al., 2064	pregnant and breastfeeding women	29.7% 06–07.2020	fear of potential consequences for their fetus/infant or themselves (respectively) resulting from vaccination during pregnancy	Strongest: Influenza vaccination in the past year, having a positive diagnosis of SARS-CoV-2, having an obstetrician following pregnancy. Others: Maternal age above 40 years, higher education, Italian as primary language.
Mohan et al., 341	pregnant and breastfeeding women	75.0% 10–11.2020	risk of infection with COVID-19, concerns about safety of the vaccine for the fetus	Strongest: Trust in safety of COVID-19 vaccine.

## Data Availability

Details of data availability are available from the first author on request.
